# High‐throughput generic single‐entity sequencing using droplet microfluidics

**DOI:** 10.1002/imt2.70087

**Published:** 2025-10-30

**Authors:** Guoping Wang, Liuyang Zhao, Yu Shi, Fuyang Qu, Yanqiang Ding, Weixin Liu, Changan Liu, Gang Luo, Meiyi Li, Xiaowu Bai, Luoquan Li, Luyao Wang, Chi Chun Wong, Yi‐Ping Ho, Jun Yu

**Affiliations:** ^1^ Institute of Digestive Diseases and Department of Medicine and Therapeutics, State Key Laboratory of Digestive Disease, Li Ka Shing Institute of Health Sciences The Chinese University of Hong Kong Hong Kong SAR China; ^2^ Department of Biomedical Engineering The Chinese University of Hong Kong Hong Kong SAR China; ^3^ Department of Electronic Engineering The Chinese University of Hong Kong Hong Kong SAR China; ^4^ Hong Kong Branch of CAS Center for Excellence in Animal Evolution and Genetics The Chinese University of Hong Kong Hong Kong SAR China; ^5^ The Ministry of Education Key Laboratory of Regeneration Medicine The Chinese University of Hong Kong Hong Kong SAR China

**Keywords:** droplet microfluidics, long‐read sequencing, metagenomics, microbial dark matter, single‐cell genomics, single‐virus genomics

## Abstract

Single‐cell sequencing has revolutionized our understanding of cellular heterogeneity by providing a micro‐level perspective in the past decade. While heterogeneity is fundamental to diverse biological communities, existing platforms are primarily designed for eukaryotic cells, leaving significant gaps in the study of other single biological entities, such as viruses and bacteria. Current methodologies for single‐entity sequencing remain limited by low throughput, inefficient lysis, and highly fragmented genomes. Here, we present the Generic Single‐Entity Sequencing (GSE‐Seq), a versatile and high‐throughput framework that overcomes key limitations in single‐entity sequencing through an integrated workflow. GSE‐Seq combines (1) one‐step generation of massive barcodes, (2) degradable hydrogel‐based in situ sample processing and whole genome amplification, (3) integrated in‐droplet library preparation, and (4) long‐read sequencing. We applied GSE‐Seq to profile viral communities from human fecal and marine sediment samples, generating thousands of high‐quality single‐entity genomes and revealing that most are novel. GSE‐Seq identified not only dsDNA and ssDNA viruses, but also hard‐to‐detect giant viruses and crAssphages. GSE‐Seq of bacterial genomes also revealed putative novel bacterial species, validating the versatility of this platform across different microbial kingdoms. Collectively, GSE‐Seq represents a robust framework that addresses persistent challenges in high‐throughput profiling for generic applications and holds immense promise for single‐cell deconvolution of diverse biological entities.

## Highlights

- Introduces Generic Single‐Entity Sequencing, a new method for high‐throughput, long‐read single‐entity sequencing.
- Utilizes degradable hydrogels for robust in situ lysis of single viruses and bacteria.
- Barcode‐guided assembly dramatically improves the recovery and completeness of viral genomes.
- Reveals vast “viral dark matter” by recovering thousands of genomes from complex samples.
- Recovers over 20,000 bacterial single‐amplified genomes (SAGs) from the human gut, including difficult‐to‐lyse species.

## Graphical abstract

We present Generic Single Entity Sequencing (GSE‐Seq), a droplet‐based workflow that generates monoclonal barcodes by one‐step PCR, performs dissolvable hydrogel‐enabled reactions for genome processing, and attaches barcodes during in‐droplet library preparation. Barcoded fragments are pooled and sequenced with PacBio long‐read sequencing. Barcodes are extracted and clustered to recover single‐entity genomes from viruses and bacteria, enabling high‐throughput single‐entity sequencing with long‐read and accurate genome reconstruction.

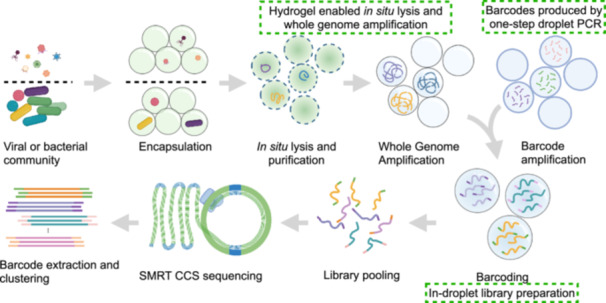

## INTRODUCTION

Single‐cell sequencing has revolutionized our understanding of cellular heterogeneity, but most existing technologies primarily focus on mammalian cells [1, 2, 3]. It remains challenging to achieve precise single‐cell genome sequencing for other biological entities, particularly for prokaryotes (e.g., bacteria, archaea) [4], virus particles [5], and organelles [6]. For many years, culture‐based methods were the go‐to approach for obtaining complete genome sequences for microbes, leaving a significant portion of uncultivable species, often referred to as “dark matter,” largely unexplored [7, 8, 9].

Metagenomic sequencing has revolutionized microbiome analyses by providing a wealth of information on previously hidden diversity of microscopic life. While metagenomics exceeds culture‐based methods in uncovering unculturable organisms, it faces two key limitations. First, while advances in long‐read sequencing have improved the continuity of metagenome‐assembled genomes (MAGs), resolving strain‐level heterogeneity remains a fundamental challenge. MAGs are computational consensus genomes that amalgamate information from multiple closely related strains, frequently obscuring subtle yet biologically critical differences in virulence factors, metabolic pathways, or phage‐host specificities [10]. Second, microbial identification depends on the availability of reference genomes, which are limited for viruses and archaea. Hence, there is an urgent need for tools providing a more comprehensive view of the microbiome.

Microfluidic technology has emerged as a powerful tool in single‐cell analyses by offering precise manipulation and analyses of individual biological entities at high throughput. Prior work, such as SiC‐seq [11] and Microbe‐seq [12], have integrated microfluidics and short‐read sequencing for the single‐cell mapping of microbiome. However, these approaches faced challenges in achieving comprehensive genome coverage and have limited applicability to microbes that were resistant to lysis. Furthermore, reliance on short‐read sequencing hampers their capability to assemble high‐quality genomes at single cell level [13, 14].

To overcome these limitations, we introduce a novel droplet microfluidics‐based platform called Generic Single Entity Sequencing (GSE‐Seq) for single‐cell microbial sequencing. GSE‐Seq enables massively parallel genome sequencing of diverse biological entities at single‐cell level, providing improved genomic coverage and resolving power as compared to conventional single‐cell microbe sequencing methods. We validated the broad applicability of GSE‐Seq in the analysis of gut and environmental microbiome at single‐cell resolution, unraveling novel bacteria and viruses at the large‐scale. Taken together, single‐cell level elucidation of the microbiome coupled to *de novo* genome assembly by GSE‐Seq has the potential to offer unprecedented functional and biological insights in diverse biological systems.

## RESULTS

### The overall design and barcode generation for generic single‐entity sequencing

**Figure 1:**
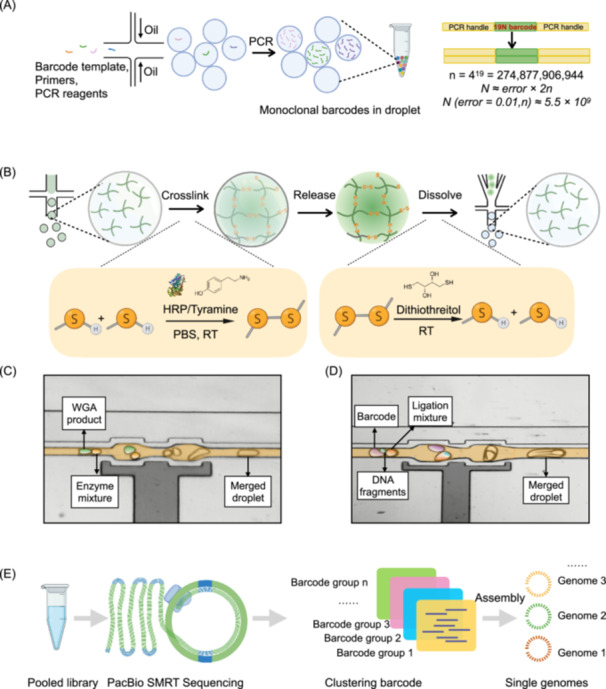
Design for high‐throughput generic single‐entity sequencing (GSE‐Seq) using droplet microfluidics. (A) A barcode pool generated by one‐step droplet PCR. Barcode templates, primers, and PCR premix are encapsulated in droplets for PCR amplification, generating monoclonal barcode clusters (left). The barcode contains 19 random bases, yielding approximately 2.7 × 10^11^ possible combinations and a barcode pool of > 5 × 10^9^, with an error tolerance < 1% (right). (B) Gelation and degradation inside droplets. The orange inset shows hydrogel crosslinking and dissociation reactions. RT: room temperature. (C) Droplets containing amplified genomes are merged with enzyme‐mixture droplets at a 1:1 ratio for simultaneous fragmentation and end‐repair. (D) Droplets containing fragmented genomes are merged with ligation‐mixture and barcode droplets at a 1:1:1 ratio for genome barcoding. (E) Workflow for obtaining genome sequences of single entities from a pooled library.

The overall design of GSE‐Seq is outlined in Figure 1. Using droplet microfluidics, we first generated millions of droplets, each containing a monoclonal barcode sequences (Figure 1A, Movie S1). Separately, the single biological entities suspended in degradable hydrogel precursor solution were individually encapsulated into droplets, followed by in‐droplet whole genome amplification (WGA) (Figure 1B), fragmentation, and end‐repair (Figure 1C). The droplets harboring single‐cell‐derived fragmented genomes were merged with barcode droplets and ligation mixture at 1:1:1 ratio, thereby labeling the individual genomes with a unique barcode. Finally, the barcoded DNA were pooled and subjected to PacBio sequencing.

The success of high‐throughput single‐cell genomics analysis hinges on high‐efficiency of barcoding. Classic barcoding strategies employ split‐and‐pool oligo synthesis on hydrogel beads, which is labor‐intensive and costly [15]. We thus developed a novel droplet barcode strategy as a low‐cost approach for generating more than billions of barcode droplets, each containing a distinct clonal population of a barcode (Figure 1A and Movie S1). Within each droplet, the PCR reagents and barcode template were mixed with three primers—a forward Primer A with a biotinylated 5'‐terminal, two reverse primers, Primer B with 5'‐terminal phosphorylation, and Primer C with a 5' adenine overhang compared to Primer B (Figure S1A and Table S1). During in‐droplet PCR amplification, the barcode oligo—a sequence of random bases flanked by two PCR handles—produces an attachable barcode. This barcode is biotinylated, double‐stranded, and features a phosphorylated end with a 3′ thymine (T) overhang on the final PCR product (Figures 1A and S1A). The 19 random bases in the barcode theoretically produce about 2.7 × 10^11^ combinations, generating a pool of > 5 × 10^9^ barcodes with a tolerance error rate of < 1% (Figure 1A). Our barcode oligos can be synthesized with a common DNA synthesizer (Table S1). With a simple one‐step in‐droplet PCR, billions of barcodes can be generated within hours.

Next, we sought to optimize droplet formulation for single‐cell encapsulation, in situ lysis, genome purification, and WGA of biological entities. One challenge is that harsh lysis conditions for bacteria and viruses are incompatible with water‐in‐oil droplets made by surfactants typically used for single‐cell sequencing. To overcome this, we have incorporated a degradable polyethylene glycol (PEG) hydrogel specifically designed to withstand these steps. This hydrogel, crosslinked via enzyme‐mediated chemistry [16], is stable during cell lysis and is designed to allow controlled degradation at later stages, thereby simplifying downstream DNA processing (Figure 1B). We encapsulated the single entities into droplets containing the hydrogel monomers. In a single run, > 100 million droplets were generated, and gelation occurred through enzyme‐mediated crosslinking at room temperature (Figure S1B−D). Following gelation, in situ lysis and DNA purification were performed within the hydrogel beads using protocols tailored for different biological entities. The hydrogel droplets containing the purified genomes were then merged with droplets containing WGA reagents and 10 mM Dithiothreitol (DTT). The DTT triggers rapid degradation of the hydrogel within 10 min (Figures S2A, S2B), releasing the captured DNA into the reaction mixture. This complete DNA release ensures high WGA efficiency, significantly outperforming methods with incomplete degradation (Figure S2C), and broadens the applicability of GSE‐Seq to diverse cell types.

The amplified DNA was then prepared for barcode ligation through in‐droplet fragmentation. Using a 2‐droplet merger, droplets containing the highly branched WGA products were merged with a custom enzyme mixture (Figure 1C, Movie S1). This mixture was designed for a sequential, two‐temperature reaction within each droplet. First, the reaction was held at 37°C, allowing T7 Endonuclease I to fragment the DNA while a Hot Start Taq DNA polymerase remained inactive. Next, the temperature was raised to 72°C to inactivate the endonuclease and activate the Taq polymerase, which adds non‐templated 3′ adenine (A) overhangs. This two‑temperature, in‐droplet reaction produces A‑tailed fragments compatible with T‐A ligation (Figure S2D).

Barcoding was then performed via in‐droplet T‐A ligation by merging these droplets with barcode droplets and ligation reagents in a precise 1:1:1 ratio using a 3‐droplet merger (Figure 1D, Movie S1). Finally, the resulting barcoded DNA molecules were pooled by breaking the emulsion, then purified and amplified to create the final sequencing library (Figure 1E).

The library design is compatible with multiple long‐read sequencing platforms, including Nanopore and PacBio [17]. For this study, the PacBio circular consensus sequencing platform was selected to generate high‐fidelity (HiFi) reads. Following sequencing, the raw HiFi reads are demultiplexed based on their unique barcodes, allowing each read to be assigned to its single entity of origin for downstream analysis (Figure 1E). This integrated workflow provides a complete solution for single‐entity sequencing, with significant advancements in efficiency, simplicity, and compatibility with diverse cell types and sequencing platforms.

### Validating the performance of GSE‐Seq

**Figure 2:**
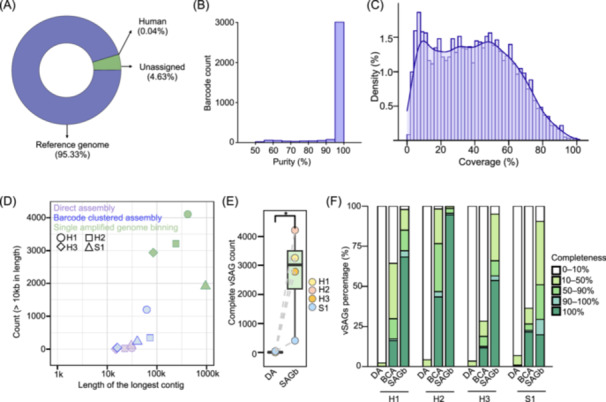
Validating the performance of GSE‐Seq. Mapping ratio (A), purity (B), and genomic coverage (C) of the viral mock community. (D) Assembly statistics for direct assembly, barcode clustered assembly, and single amplified genome binning. H1–H3, three human fecal viral samples; S1, a marine sediment sample. (E) Numbers of genomes (completeness = 100%) obtained using different assembly methods. The data were analyzed by a two‐tailed paired *t*‐test; *p < 0.05. (F) Completeness distribution of fecal and marine viral genome assemblies using different methods. BCA, barcode clustered assembly; DA, direct assembly; GSE‐Seq, Generic Single Entity Sequencing; SAGb, single amplified genome binning.

To validate the performance of GSE‐Seq, we analyzed a mock viral community composed of three *Escherichia coli* bacteriophages: T4, P1, and Lambda. The resulting single amplified genome (SAG) library produced HiFi reads with 96.5% of reads ≥ Q30 and read N50 of 1.7 kb, approximately six times longer than that of current short‐read methods (Figure S3A). Over 95% of total reads aligned to the three phage reference genomes, with minimal residual reads likely originating from environmental contamination (Figure 2A).

From these reads, we identified 3564 barcode clusters, each corresponding to a viral SAG (vSAG) (Figure S3B). The method demonstrated excellent purity: 98.8% of these clusters (3523/3564) showed less than 1% external contamination. Furthermore, a total of 3036 vSAGs (85.2%) were classified as high‐purity, with over 95% of reads mapping to a single reference phage (Figure 2B and Table S2). The relative abundance of vSAGs for each phage matched qPCR measurements, indicating no significant quantification bias was introduced (Figure S3C). In terms of genome coverage, 23 vSAGs achieved > 90% completeness, and 998 vSAGs (32.8%) ranged from 50% to 90% complete (Figure 2C). We observed uneven coverage in certain genomic regions (Figure S3D), which may be attributed to the inherent biases of WGA (Figure S3D). Collectively, these results confirm that GSE‐Seq generates high‐purity, high‐coverage vSAGs from a defined community.

Genome assembly in complex communities remains a persistent challenge. Conventional metagenomic assembly often results in mis‐assemblies and fragmented contigs mainly due to consensus sequences. GSE‐Seq helps resolve these issues by segregating reads to single‐cell level before assembly. We evaluated three strategies using viral communities from three human fecal samples (H1, H2, H3) and one marine sediment sample (S1). These strategies included direct assembly (DA), where all reads are pooled; barcode‐clustered assembly (BCA), which first segregates reads by barcode; and single amplified genome binning (SAGb), which assembles and bins reads within each barcode cluster. Both barcode‐aware methods significantly outperformed DA in generating longer contigs (Figures 2D and S4A). Compared to DA, BCA increased the maximum contig length by an average of 1.9‐fold and yielded 23.6 times more contigs > 2000 bp and 15.1 times more contigs > 10,000 bp (Figures S4B–D and Table S3). The SAGb strategy offered even more dramatic improvements, increasing the maximum contig length by 15.2‐fold and producing 14.4‐fold increase in contigs > 2000 bp and an exceptional 214‐fold increase in contigs > 10,000 bp (Figure S4B–D and Table S3).

We next evaluated the completeness of viral genome assembly (i.e., the proportion of the expected genome recovered) using CheckV [18]. The number of complete vSAGs (completeness = 100%) was significantly higher using SAGb (*p* < 0.05) compared to DA (Figure 2E and Table S4). Whereas most vSAGs derived from DA exhibited a low degree of completeness (< 10%), BCA and SAGb consistently generated vSAGs with > 50% completeness (Figures 2F and S4E). In human fecal samples, SAGb delivered more complete viral genomes compared to BCA, whereas they performed similarly in the marine sample (Figures 2E,F and S4E).

To confirm these benefits on larger genomes, we applied the same assembly strategies to gut bacteria from a human sample (H4). Consistent with the viral results, both BCA and SAGb greatly enhanced the assembled contig length and the number of long contigs (> 2000 bp and > 10,000 bp) compared to DA (Figure S4B−D and Table S3). Collectively, these findings demonstrate that partitioning complex metagenomic reads at the single‐cell level is a powerful strategy for improving genome assembly, a benefit that is further enhanced by barcode binning.

### GSE‐Seq uncovers vast viral diversity in the human gut and marine sediment

**Figure 3:**
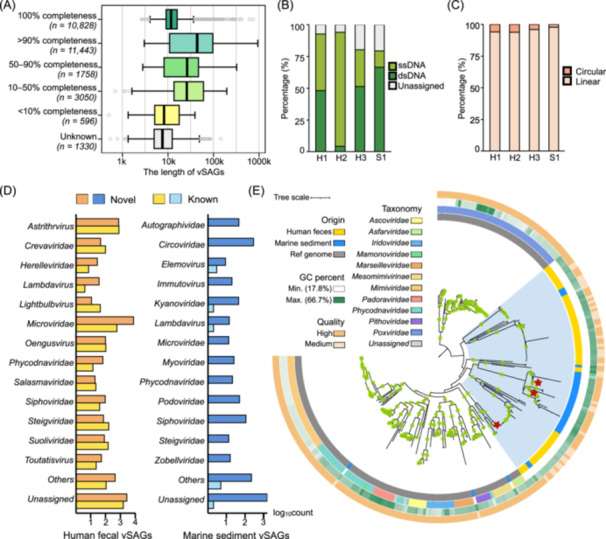
GSE‐Seq uncovers vast viral diversity in the human gut and marine sediment. (A) Distribution of estimated genome completeness and length of viral single amplified genomes (vSAGs) into quality tiers. (B) Baltimore classification of vSAGs from human feces and marine sediment. (C) Complete circular genomes detected by GSE‐Seq. (D) Distribution of novel vSAGs across different families or floating genera. (E) Maximum‐likelihood tree of 100 putative nucleocytoplasmic large DNA viruses (NCLDV) vSAGs together with isolated NCLDV reference genomes. Tree annotations from inside to the outside: (1) origin, (2) taxonomy, (3) GC content, and (4) genome quality. Green dots on branches represent bootstrap support ≥70%. The blue shading marks the main clades containing the putative NCLDV vSAGs recovered in this study. Red stars denote three vSAGs with genomic structures shown in Figure S5A.

Current single virus sequencing [5] relies on fluorescence–activated cell sorting to isolate individual viral particles followed by library construction, which is labor‐intensive and low‐throughput [19, 20]. In contrast, our GSE‐Seq platform with the SAGb assembly strategy enables a massive increase in throughput. From the fecal samples of three healthy donors, we recovered a total of 15,710 viral single amplified genomes (vSAGs), and from a marine sediment sample, we recovered another 2467 vSAGs (Tables S5, S6). The quality of these genomes was exceptionally high: 59.6% (10,828) demonstrated 100% completeness, and 63.0% (11,443) showed greater than 90% completeness (Figure 3A). The recovered vSAGs included a diverse mix of dsDNA, ssDNA, linear, and circular forms. Notably, ssDNA viruses constituted 52.2% of the human gut vSAGs and 12.8% of the marine vSAGs (Figure 3B). Furthermore, we identified complete circular viral genomes, accounting for 5.4% and 2.2% of the fecal and marine vSAGs, respectively (Figure 3C).

While viral metagenomics has expanded our knowledge of viral diversity, 60%−95% of viruses remain uncharacterized, earning them the moniker “dark matter” [21]. Our results confirm this on a single‐genome level. Only 73.1% of human fecal vSAGs and 36.5% of marine vSAGs could be taxonomically classified at family or free‐floating genus levels (Figure 3D and Table S5). When compared to publicly available databases, the novelty was even more striking: only 23.1% of gut vSAGs and a mere nine marine vSAGs (0.36%) matched any known species in the NCBI RefSeq or Global Ocean Viromes 2.0 (GOV 2.0) databases [22] (Figure 3D and Table S6). This low match rate in the marine sample may be partly due to the underrepresentation of the seas around East Asia in the GOV 2.0 database. Together, these findings highlight a largely untapped viral diversity and demonstrate the power of GSE‐Seq to explore it.

GSE‐Seq also proved effective at recovering nucleocytoplasmic large DNA viruses (NCLDVs), an aspect often overlooked by conventional metagenomics due to their large genome size and scarcity [23]. We identified 100 vSAGs belonging to the NCLDVs based on the presence of at least two *Nucleocytoviricota* marker genes. The largest of these genomes was 948 kb (Table S7). Only a single vSAG exhibited > 70% average amino acid identity (AAI) and > 20% coverage with the reference genomes (Table S7). Excluding three vSAGs, which were placed within *Mesomimivirinae, Marseilleviridae*, and *Phycodnaviridae*, the majority of NCLDV SAGs were clustered into independent clades with no isolate representatives (Figure 3E). Our identified NCLDV vSAGs showed a considerable divergence from existing NCLDVs cataloged in the Giant Virus Database [24]. Functional annotation of these putative NCLDV genomes revealed an abundance of genes involved in core metabolic and informational processes, including replication, transcription, and translation (Figure S5A and Table S8).

In addition, we recovered numerous crAssphages, a highly abundant but heterogeneous phage group that is challenging to assemble, which was first discovered in 2014 using cross assembly [25]. We detected 92 crAssphage‐like vSAGs in sample H1 and 88 in sample H3, identified by the presence of at least two conserved hallmark genes [26] (Table S9). In summary, GSE‐Seq allows high‐throughput, deep mining of human and marine virome, proving highly effective for the discovery of novel viruses and the recovery of diverse, hard‐to‐detect viral groups.

### GSE‐Seq uncovers novel bacterial diversity in the human gut

**Figure 4:**
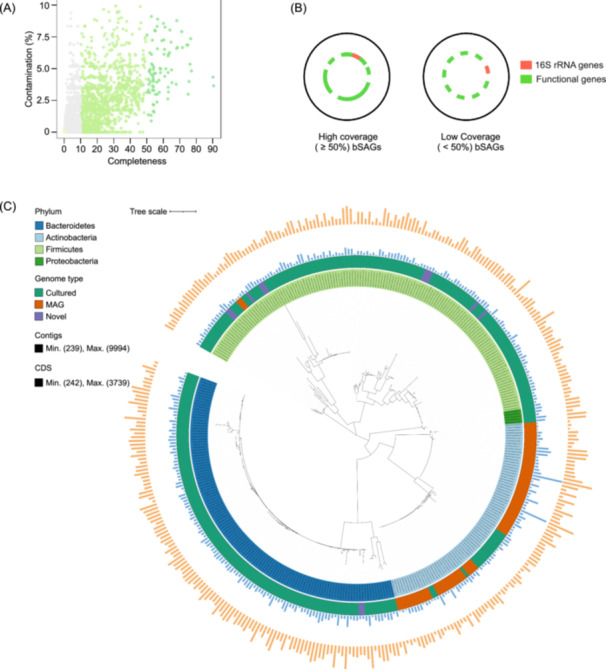
Application of GSE‐Seq to bacterial communities from the human gut. (A) Completeness of bacterial single amplified genome assemblies (bSAGs). (B) Conceptual illustration of the value from bSAGs with varying coverage. High‐coverage bSAGs (≥50%) enable substantial genome reconstruction, whereas low‐coverage bSAGs (<50%), though incomplete, physically link a taxonomic marker such as the 16S rRNA gene (red) to other functional genes (green) from the same single cell, providing deeper biological context than standalone 16S amplicon sequencing. (C) Taxonomic annotation of the identified bSAGs. In total, 360 bSAGs contain near‐complete 16S rRNA sequences (> 1000 bp). These bSAGs are classified into 4 phyla, 6 classes, 6 orders, 9 families, and 27 genera. Tree annotations from inside to the outside: (1) bSAGs colored by phylum, (2) genome type according to the UHGG database, including cultured strains, metagenome‐assembled genomes (MAGs), and putative novel genomes, (3) contig lengths, and (4) CDS counts. GSE‐Seq, Generic Single Entity Sequencing.

To determine the effectiveness of GSE‐Seq on microbes with larger genomes and rigid cell walls, we conducted single bacteria sequencing of a human fecal sample. In total, we recovered 23,266 bacterial single amplified genomes (bSAGs). The quality of these genomes varied, with two bSAGs achieving > 90% completeness, 81 classified as medium‐quality (50%–90% completeness; quality score ≥ 10), and 1210 as draft‐quality (10%–50% completeness) (Figure 4A and Table S10). The 83 medium‐ and high‐quality bSAGs belonged to the dominant human gut phyla—Actinobacteria, Firmicutes, and Bacteroidetes (Figure S5B and Table S11) [27]. Among these, 51 bSAGs (61.4%) represented putative novel species based on Average Nucleotide Identity (ANI) analysis, while 31 were assigned to known species, *Bifidobacterium longum*, *Dorea formicigenerans*, *Gemmiger formicilis*, and *Prevotella copri* (Table S11). This high proportion of novelty is consistent with estimates that over 70% of human gut microbial species remain unannotated [28].

Due to the larger size of bacterial genomes, genome coverage was less comprehensive than for viruses. Nevertheless, the bSAGs serve as a valuable bridge between 16S rRNA gene surveys and metagenomics (Figure 4B). We identified 360 bSAGs containing near‐complete 16S rRNA gene sequences (> 1000 bp), which spanned 4 phyla, 6 classes, 6 orders, 9 families, and 27 genera (Figure 4C and Table S12). Our 16S rRNA gene‐based phylogenetic analysis revealed that 22.2% of the analyzed samples had lower than 97% similarity to the 16S sequences of cultured species, suggesting they represent potentially novel species. Notably, we also recovered several Gram‐positive genera that are typically resistant to lysis, including *Bifidobacterium, Collinsella, Clostridium XIVa, Dysosmobacter, Faecalibacterium, Lachnospira, Oscillibacter, Ruminococcus*, and *Ruminococcus2*, suggesting the potential of our method for studying these challenging microbes (Table S12).

GSE‐Seq's combination of long‐read sequencing and single‐bacterium resolution is a potent tool for identifying prophages, which are difficult to resolve from host genomes using short‐read metagenomics [29]. From the bSAGs, we identified 1494 high‐quality prophage‐host pairs (Table S13). Remarkably, 97% of these prophages were classified under *Caudoviricetes*, the predominant class of bacteria‐infecting viruses. Our analysis also revealed specific high‐frequency interactions, such as a 28.1% interaction rate for *Prevotella sp015074785* (Figure S5C). This ability to capture host‐phage linkages at the single‐cell level provides a powerful tool for studying viral infections and coevolutionary dynamics.

We next performed functional annotation of the recovered SAGs. In the vSAGs, we identified key viral genes related to structure, such as capsid and tail collar domain proteins (Table S14 for gut‐derived vSAG; Table S15 for marine sediment‐derived vSAG). We annotated 4.7 million genes in bSAG, revealing pathways for sugar metabolism and amino acid biosynthesis. We also identified 1908 antibiotic resistance genes, conferring resistance to compounds such as glycopeptides, tetracyclines, and fluoroquinolones (Table S16).

## DISCUSSION

The Earth's biosphere is home to an immense, yet largely untapped, diversity of life. Traditional methods, whether culture‐based or metagenomic analysis, often fail to capture this diversity, particularly when distinguishing novel, closely related, or low‐abundance microbes. Our results demonstrate that GSE‐Seq directly addresses these challenges by enabling the high‐throughput sequencing of individual genomes. We validated its performance by unraveling novel viruses and bacteria from human fecal samples and marine sediment, demonstrating the broad applicability of this technology and its capacity to identify hard‐to‐detect species like NCLDVs, crAssphages, and prophages.

GSE‐Seq's integrated workflow distinguishes it from other recently developed single‐cell genomic methods. For instance, while DoTA‐seq is a valuable tool for targeted analysis, it does not perform whole‐genome sequencing [30]. The recently described EASi‐seq platform enables single‐cell whole‐genome analysis but is based on short‐read sequencing, which can limit assembly completeness [31]. In contrast, GSE‐Seq is the first platform to integrate high‐throughput single‐cell partitioning with PacBio long‐reads, enabling superior genome assembly. Furthermore, our one‐step droplet PCR for barcode generation offers a highly scalable and cost‐effective strategy, making high‐throughput single‐cell genomics more accessible.

A cornerstone of GSE‐Seq's versatility is its use of dissolvable PEG hydrogel beads for robust, in‐droplet sample processing. This approach addresses a key bottleneck in single‐entity sequencing: the need for effective lysis across diverse cell and capsid types. Unlike methods using porous agarose matrices that require heat and are difficult to degrade [11], our PEG hydrogel solidifies at room temperature and is designed for controlled degradation with DTT. This critical feature allows single entities to be subjected to harsh chemical and enzymatic treatments essential for robustly lysing diverse viral capsids and tough bacterial cell walls—conditions that would compromise other systems. The subsequent complete dissolution of hydrogel ensures maximal DNA recovery and compatibility with downstream WGA and library preparation, leading to improved detection and genome quality.

Here, we demonstrated the capability of GSE‐Seq to decipher a mock viral community, and complex real‐world samples. By using barcode‐based partition of sequencing data into individual virus before *de novo* genomic assembly, we achieved substantially improved contig length and genome completeness, including complete circular viral genomes. Remarkably, a substantial proportion of vSAGs generated from human fecal samples and especially marine sediment showed no significant similarity to publicly existing databases, suggesting that GSE‐Seq is a powerful tool for exploring viral “dark matter”. GSE‐Seq also showed exceptional ability to scan hard‐to‐detect viruses, including NCLDVs and crAssphages that are not readily detected by conventional metagenomic approaches.

GSE‐Seq addresses shortcomings of pseudo‐metagenomic assembly. Even with long‐read PacBio sequencing, the combined effects of suboptimal read lengths (~1.8 kb), WGA‐induced fragmentation and bias, and complexity of viral communities lead to poor performance of pseudo‐metagenomic assembly. By barcoding single viruses, our method dramatically improves the accuracy and completeness of genome reconstruction. This single‐cell granularity is invaluable for identifying rare or unculturable species and exploring microbial heterogeneity in unprecedented detail, offering a powerful complementary approach to conventional metagenomics.

We extended the application of GSE‐Seq in the profiling of single bacteria and virus in the human gut, confirming its broad applicability in single‐cell analysis. While the recovery rate of high‐quality bacterial genomes was modest compared to deep metagenomics, the primary advantage of GSE‐Seq is its unparalleled resolution. By linking genomic data to a single cell of origin, GSE‐Seq can resolve strain‐level heterogeneity and accurately identify phage‐host interactions that are obscured in consensus‐based assemblies. This capability is crucial for applications such as identifying candidates for phage therapy and deciphering microbiome‐immune interactions.

We envision that our approach can be optimized for other microbial identities. For instance, archaea, with their unique cell envelope structures, might require additional lysis reagents such as strong detergents and archaeal‐specific enzymes [32, 33]. Our PEG hydrogel beads could withstand very harsh lysis conditions and accommodate these protocols with minimal optimization. In light of its ability to generate viral genomes with high completeness, future work could incorporate a one‐step reverse transcription process to broaden the use of GSE‐Seq to explore immense and underexplored diversity of RNA viruses [34]. Future studies will examine GSE‐Seq's applications across diverse eukaryotic genomes, including those from plant, insect, and protist single cells. These can undoubtedly enhance the scope of GSE‐Seq and its ability to probe the vast and unexplored biological realm.

Although our mock community analysis demonstrated high purity and low contamination, we acknowledge several areas for improvement. A formal benchmark using a mock bacterial community is necessary to fully validate performance on bacteria. Technical refinements, such as reducing DNA leakage from hydrogel beads and employing more uniform WGA methods like primary template‐directed amplification [35] or linear amplification via transposon insertion (LIANTI) [36], could further enhance genome coverage and quality. We also acknowledge that a direct quantitative comparison with matched metagenomic sequencing on complex samples would be required to fully assess recovery biases, representing an important area for future validation. Furthermore, while our method is theoretically well‐suited for strain‐level resolution assessment, a formal demonstration of this capability requires further research.

Sequencing depth is critical for improving GSE‐Seq performance. GSE‐Seq combines single‐cell partitioning to reduce assembly complexity and long‐read platform spanning repetitive regions and structural variations, thus improving continuity and completeness of microbial genomes. While third‐generation sequencing (e.g., PacBio, Nanopore) provides long reads, combining long‐read single‐cell sequencing with next‐generation sequencing could generate a high volume of short reads to assist in genome assembly. In future studies, this hybrid approach to integrate metagenomic and SAGs may improve both continuity and completeness of larger genomes, such as those from bacteria and larger viruses [13, 37, 38, 39, 40].

## CONCLUSION

GSE‐Seq represents a significant step towards capturing the vast biological diversity. By integrating ultrahigh‐throughput barcode generation, robust hydrogel‐based sample processing and WGA, in‐droplet library preparation, and long‐read sequencing, we achieved the single‐cell profiling of microbial genomes, yielding novel viral and bacterial species in human fecal and marine samples. Ultimately, the versatility of GSE‐Seq provides a powerful and accessible framework for exploring single‐entity genomics across diverse biological systems.

## METHODS

### Sample collection and preparation

The study utilized a three‐phage mock viral community consisting of *Escherichia coli* bacteriophages T4 (DSM4505), P1 (DSM5757), and Lambda (DSM4499). Complex real‐world samples included human fecal samples from four healthy donors (H1–H4) and a marine sediment sample collected from Hong Kong, China (22°18′59.1″N, 113°56′41.2″E). Viral‐like particles (VLPs) were extracted and purified from these samples following a modified version of a previously published protocol [41]. For bacterial analysis, cells were isolated from the fresh stool sample of donor H4. Detailed protocols for VLP and bacterial cell purification are available in the Supplementary Method.

### GSE‐Seq platform and workflow

The GSE‐Seq workflow was conducted using five custom‐designed microfluidic devices fabricated via soft lithography (Figure S6, details in Supplementary Method). The core workflow proceeds as follows:

Barcode Generation: A library of over 10^9^ unique barcodes was generated in a single run using a one‐step in‐droplet PCR method. The reaction mixture contained a barcode template, a custom primer mix (Table S1), and TspDNA polymerase (Invitrogen, Cat. no. 11448‐024).

Single‐Entity Encapsulation and Lysis: Single viral or bacterial entities were encapsulated into droplets containing a degradable hydrogel solution composed of 4‐arm PEG‐20k‐SH (Sinopeg, Cat. no. 06020701312). Following overnight gelation, in‐situ lysis was performed. Viral lysis was achieved using Viral DNA Buffer (Zymo Research, Cat. no. D3016‐1). Bacterial lysis was performed using an enzymatic cocktail including MetaPolyzyme (Sigma‐Aldrich, Cat. no. MAC4L‐5MG) and Proteinase K (Invitrogen, Cat. no. 100005393), followed by treatment with a chaotropic buffer.

In‐Droplet WGA and Library Preparation: After lysis and purification, the hydrogel beads were re‐encapsulated into new droplets with an MDA pre‐mix (Whole Genome Amplification Kit, 4basebio, Cat. no. 380100) and Dithiothreitol (DTT) to dissolve the hydrogel, releasing the genomic DNA for amplification. Following amplification, the DNA was fragmented and end‐repaired in‐droplet using T7 Endonuclease I (NEB, Cat. no. M0302L) and Hot Start Taq DNA Polymerase (NEB, Cat. no. M0495L). Barcoding was then achieved in a three‐droplet‐merger device, where the repaired genomic fragments were ligated to unique barcodes using the Ultra II Ligation Master Mix (NEB, Cat. no. E7648A).

### SMRT sequencing

The pooled and purified barcoded libraries were prepared for sequencing using the SMRTbell Express TPK 2.0 kit (PacBio) and sequenced on a PacBio Sequel II platform to generate high‐fidelity (HiFi) long reads.

### Bioinformatic analysis

High‐fidelity (HiFi) reads were generated from the raw PacBio data using ccs (v6.0.0) with parameters of at least three passes and a predicted accuracy of ≥0.90. A custom Python script and starcode (v1.4) were then used to extract barcodes and demultiplex the reads into individual single amplified genomes (SAGs).

For genome assembly, we compared three strategies: direct assembly (DA), BCA, and SAGb, all performed with SPAdes (v3.13.0). The quality and completeness of the resulting viral SAGs (vSAGs) were assessed with CheckV (v0.8.1), while bacterial SAGs (bSAGs) were assessed with CheckM (v1.2.0).

Taxonomic classification was performed using the Contig Annotation Tool (v4.6) against the NCBI nonredundant (nr) protein database. Genome novelty was determined by calculating the ANI against public databases (NCBI RefSeq, MGV, GVD, and GOV v2.0) using FastANI (v1.33). Functional annotation of predicted genes was conducted using eggNOG‐mapper (v2).

Specialized pipelines were used to identify key viral taxa. NCLDVs were predicted using RBS classifier and ViralRecall (v2.0), and subsequently confirmed by marker gene presence. crAss‐like phages were identified by predicting open reading frames with Prodigal (v2.6.3) and searching for hallmark viral genes with hmmscan (v3.3.0). Prophages were detected using CheckV (v0.8.1) and VirSorter2 (v2.2.3). Functional annotation was conducted using eggNOG‐mapper (v2).

Detailed descriptions of all bioinformatic pipelines and parameters are available in the Supplementary Method.

## AUTHOR CONTRIBUTIONS

**Guoping Wang**: Conceptualization; Investigation; methodology; data curation. **Liuyang Zhao**: Conceptualization; investigation; methodology; data curation. **Yu Shi**: Investigation; methodology; data curation. **Fuyang Qu**: Investigation; methodology. **Yanqiang Ding**: Formal analysis. **Weixin Liu**: Formal analysis. **Changan Liu**: Investigation. **Gang Luo**: Investigation. **Meiyi Li**: Investigation. **Xiaowu Bai**: Investigation. **Luoquan Li**: Investigation. **Luyao Wang**: Formal analysis. **Chi Chun Wong**: Writing—review and editing. **Yi‐Ping Ho**: Resources; supervision; formal analysis; methodology; writing—review and editing. **Jun Yu**: Conceptualization; methodology; supervision; funding acquisition; writing—original draft; writing—review and editing; project administration; resources. All authors have read the final manuscript and approved it for publication.

## CONFLICT OF INTEREST STATEMENT

Guoping Wang, Liuyang Zhao, Fuyang Qu, Yi‐Ping Ho, and Jun Yu are the inventors in the pending patent application related to this study.

## ETHICS STATEMENT

This study was approved by the Joint Chinese University of Hong Kong‐New Territories East Cluster Clinical Research Ethics Committee (CREC Ref. No.: 2018.359).

## Supporting information


**Figure S1.** Detailed design for GSE‐Seq.
**Figure S2.** Characterization of hydrogel degradation and in‐droplet enzymatic reactions.
**Figure S3.** Quality assessment and sequencing metrics of the viral mock community.
**Figure S4.** Comparison of genome assembly statistics across different methods.
**Figure S5.** Functional, phylogenetic, and host‐linkage analysis of viral and bacterial SAGs.
**Figure S6.** Microfluidic chip designs for the GSE‐Seq workflow.

**Table S1.** Oligonucleotides used in this study.
**Table S2.** Quality metrics of viral single amplified genomes (vSAGs) from the mock viral community.
**Table S3.** Comparison of assembly statistics for Direct assembly (DA), Barcode‐clustered assembly (BCA), and Single Amplified Genome binning (SAGb).
**Table S4.** Comparison of viral genome completeness for Direct assembly (DA), Barcode‐clustered assembly (BCA), and Single Amplified Genome binning (SAGb).
**Table S5.** Taxonomic classification and characteristics of viral single amplified genomes (vSAGs) from human fecal samples.
**Table S6.** Taxonomic classification and characteristics of viral single amplified genomes (vSAGs) from the marine sediment sample.
**Table S7.** Characteristics and annotation of Nucleocytoplasmic Large DNA Virus (NCLDV) single amplified genomes (vSAGs).
**Table S8.** COG functional annotation of predicted genes in Nucleocytoplasmic Large DNA Virus (NCLDV) single amplified genomes (vSAGs).
**Table S9.** Characteristics of crAssphage single amplified genomes (vSAGs) identified in human fecal samples.
**Table S10.** Quality metrics and taxonomic classification of bacterial single amplified genomes (bSAGs) from a human fecal sample.
**Table S11.** Characteristics and annotation of medium‐ and high‐quality bacterial single amplified genomes (bSAGs).
**Table S12.** Taxonomic classification of bacterial single amplified genomes (bSAGs) based on 16S rRNA gene sequences.
**Table S13.** Predicted prophage‐host pairings identified from bacterial single amplified genomes (bSAGs).
**Table S14.** Functional annotation of predicted genes from viral single amplified genomes (vSAGs) in human fecal samples.
**Table S15.** Functional annotation of predicted genes from viral single amplified genomes (vSAGs) in the marine sediment sample.
**Table S16.** Functional annotation of predicted genes from bacterial single amplified genomes (bSAGs).
**Table S17.** Sequencing and barcode clustering statistics for all GSE‐Seq experiments.

**Movie S1.** Real‐time visualization of key microfluidic steps in the GSE‐Seq Workflow.

## Data Availability

The data that support the findings of this study are openly available in Figshare at https://figshare.com/s/1d3869d01de5853a6268. The customized code of analysis is available on GitHub at the following URL: https://github.com/Guoping-Wang/GSE_seq. Supplementary materials (methods, figures, tables, graphical abstract, slides, videos, Chinese translated version, and update materials) may be found in the online DOI or iMeta Science http://www.imeta.science/.
