# Supplementary material for: High‐throughput generic single‐entity sequencing using droplet microfluidics
Source: Imeta. 2025 Oct 30;4(6):e70087. doi: 10.1002/imt2.70087 (PMC12747555; doi:10.1002/imt2.70087)
Supplement: Supplementary file 1 — Figure S1. Detailed design for GSE‐Seq. Figure S2. Characterization of hydrogel degradation and in‐droplet enzymatic reactions. Figure S3. Quality assessment and sequencing metrics of the viral mock community. Figure S4. Comparison of genome assembly statistics across different methods. Figure S5. Functional, phylogenetic, and host‐linkage analysis of viral and bacterial SAGs. Figure S6. Microfluidic chip designs for the GSE‐Seq workflow. [file IMT2-4-e70087-s003.docx]

Supporting information to

**High-throughput generic single-entity sequencing using droplet microfluidics**

**Running title**: GSE-Seq for single-entity sequencing

Guoping Wang^1#^, Liuyang Zhao^1#^, Yu Shi^1#^, Fuyang Qu^2#^, Yanqiang Ding^1#^, Weixin Liu^1^, Changan Liu^1^, Gang Luo^3^, Meiyi Li^1^, Xiaowu Bai^1^, Luoquan Li^2^, Luyao Wang^1^, Chi Chun Wong^1^, Yi-Ping Ho^2,4,5^*, Jun Yu^1^*

^1^Institute of Digestive Diseases and Department of Medicine and Therapeutics, State Key Laboratory of Digestive Disease, Li Ka Shing Institute of Health Sciences, The Chinese University of Hong Kong, Hong Kong SAR, China

^2^Department of Biomedical Engineering, The Chinese University of Hong Kong, Hong Kong SAR, China

^3^Department of Electronic Engineering, The Chinese University of Hong Kong, Hong Kong SAR, China

^4^Hong Kong Branch of CAS Center for Excellence in Animal Evolution and Genetics, The Chinese University of Hong Kong, Hong Kong SAR, China

^5^The Ministry of Education Key Laboratory of Regeneration Medicine, The Chinese University of Hong Kong, Hong Kong SAR, China

^#^These authors contributed equally: Guoping Wang, Liuyang Zhao, Yu Shi, Fuyang Qu, Yanqiang Ding.

*Correspondence: [junyu@cuhk.edu.hk](mailto:junyu@cuhk.edu.hk) (Jun Yu), [ypho@cuhk.edu.hk](mailto:ypho@cuhk.edu.hk) (Yi-Ping Ho)

**METHOD**

Fabrication and assembly of the microfluidic devices

All chips (Figure S6) in our study were fabricated following the widely used soft lithography process, as previously described [1,2]. Our methodology involved five microfluidic chips, each designed for a specific purpose: single particle isolation, whole-genome amplification, debranching of multiple displacement amplification (MDA) products, barcode generation, and genomic DNA barcoding. The designs for the microfluidic devices were created using AutoCAD software (Autodesk, San Rafael, US) and subsequently fabricated through soft lithography. Photomasks required for the process were printed at a resolution of 25,400 dots per inch (DPI) by MicroCAD Photomask Ltd. (Shenzhen, China).

The fabrication process began with the preparation of silicon wafers. The SU-8 3050 photoresist (Kayaku Advanced Materials, Westborough, MA, USA) was applied to a silicon wafer and crosslinked by baking at 120 °C for 30 min. The coated wafer was then exposed to UV light through the respective photomasks. The next stage involved fabricating microfluidic devices. Polydimethylsiloxane (PDMS; prepolymer-to-curing agent ratio = 10:1, Dow Corning, MI, USA) was poured over the silicon wafer patterned with the SU-8 3050 photoresist. The PDMS devices were cured in an oven at 80 °C overnight, carefully extracted from the wafer using a metal scalpel, and punctured with 1.0-mm holes to create the inlets and outlets. The devices were cleaned sequentially with 100% ethanol and ultrapure distilled water, then blow-dried with nitrogen gas. The PDMS slabs were bonded to glass coverslips using semi-cured PDMS (prepolymer-to-curing agent ratio = 2:1) and baked at 80 °C overnight. Finally, the channels were treated with Aquapel and baked at 80 °C for 10 min to render them hydrophobic.

Preparation of viral samples

The performance of GSE-Seq was validated using the following three *Escherichia coli* bacteriophages: T4 (DSM4505), P1 (DSM5757), and Lambda (DSM4499). These phages were propagated on their respective host strains according to DSMZ protocol. Subsequently, phage lysates were centrifuged at 3000 × g for 15 min, and the resulting supernatants were filtered twice through a 0.22-μm membrane. These filtrates were then concentrated using an Amicon 50-kDa-molecular-mass Amicon Ultra-15 Centrifugal Filter (Millipore, no. UFC905024), washed twice with 15 ml SM buffer (200 mM NaCl, 8 mM MgSO_4_, 50 mM Tris-HCl, 0.01% Gelatin, pH 7.5), and concentrated to a final volume of approximately 500 μl. The viral concentrates were treated with Benzonase endonuclease (250 U/ml; Sigma-Aldrich, no. E8263) at 4℃ for 8 to 12 h to degrade free nucleic acids. The activity of Benzonase endonuclease was subsequently inhibited by the addition of 10 mM EDTA. The resulting viral-like particles (VLPs) suspended in SM buffer can be stored at 4 ℃ for up to 3 months. Because virus quantification by SYBR Gold staining and fluorescence microscopy varies in accuracy, the effective mixing ratio of the phages was determined by qPCR analysis, revealing a ratio of 1000:500:1 (T4:P1:Lambda) in the mock community.

Our GSE-Seq process was applied to stool samples from three healthy individuals (H1, a 27-year-old male; H2, a 27-year-old female; and H3, a 29-year-old male) and marine sediment (S1) collected in Hong Kong, China (22°18'59.1"N, 113°56'41.2"E, -8.756 m). VLP extraction and purification were performed following a modified version of a previously published protocol [3]. Immediately after collection, stool samples were homogenized in SM buffer with a Bioprep-24 instrument at a velocity of 6 m/s, processed twice for 45 s each with a 5-min rest between cycles. The homogenates were then centrifuged at 10,000 × g for 5 min. The marine sediment, kept on ice during transport and stored at -80 °C until processing, was homogenized in SM buffer through vertexing for 5 min and centrifuged at 4000 × g for 10 min to gather the supernatant. Subsequently, the derived supernatants from fecal or marine samples were sequentially filtered through 20-μm and 5-μm mesh filters and followed by triple filtration through a 0.22-μm filter. The resulting virus suspensions were concentrated, and free nucleic acids were removed using the same procedure applied to phage cultures.

Purified VLPs were diluted in SM buffer and filtered onto 0.02-μm Anodisc polycarbonate filter (Whatman, no. 6809-6002). The filters were then stained with 4 × SYBR Gold (Thermo Fisher Scientific, S11494) for 15 min and rinsed with ultra-pure H_2_O. Once dried, each filter was positioned on a glass slide using 15 μl of antifade fluorescence mounting medium (Abcam, ab104135). SM buffer alone was used as a blank control. Filters were examined under an epifluorescence microscope within 12 h filtration. Viruses were enumerated across 4 to 6 randomly chosen fields of view (50 × 50 μm each) per filter. Viral counts per ml of sample were calculated according to a previously established protocol [4].

Preparation of bacterial samples

Fresh stool samples from a healthy donor (H4, a 29-year-old male) were collected and processed. The sample was homogenized using a Bioprep-24 Homogenizer at a velocity of 4.5 m/s, applied five times for 45 s each with 1-minute cooling periods on ice between runs. After homogenization, the mixture was centrifuged at 500 g for 2 min to remove large debris, followed by sequential filtration using cell strainers with pore sizes of 70 µm, 40 µm, 20 µm, and 10 µm. The bacteria-rich solution was centrifuged at 5000 g for 5 min, yielding a pellet washed three times with pre-chilled 1× PBS to reduce contamination from non-bacterial particles, extracellular DNA, and potential inhibitors of downstream processes. The resultant bacteria were stained using 1 × SYBR Green, and their concentration was estimated by counting the fluorescent entities under a microscope.

Single particle isolation

Single entities were encapsulated with a hydrogel solution within droplets of about 22 µm in diameter. The concentration was estimated by the Poisson distribution [5]. Hydrogel solution preparation, undertaken on ice, entailed resuspending particles in a 1 ml mixture of 1 × PBS, 4-arm PEG-20k-SH (2.5% for VLPs, 5% for bacteria), 17% Optiprep Density Gradient Medium, 5 mM Tyramine, and 1 μl of HRP. During droplet generation, the hydrogel solution-containing syringe was kept cool with ice-water packs, with flow rates set at 2-2.5 µl/min for particle suspension and 8-10 µl/min for Droplet Generation Oil (Bio-Rad, no. 1864006). The resulting droplets were collected and incubated at 25 °C with shaking at 1000 rpm overnight to allow PEG gel beads to crosslink.

Lysis inside gel beads

Following gelation, droplet emulsions were washed twice with 100% isopropanol containing 0.1% Triton X-100 and centrifuged at 50 × g for 1 min to pellet the gel beads. This was followed by two washes with 70% ethanol containing 0.1% Triton X-100 and a 2-minute centrifugation at 1000 × g. Microgels were washed three times with TE buffer containing 0.1% Tween-20 and centrifuged for another 2 min. The resulting microgel pellets could be either processed immediately or stored in TE buffer containing 0.1% Tween-20. To lyse VLPs within gel beads, a four-fold volume of Viral DNA Buffer (Zymo Research, no. D3016-1) was added, followed by a 30-minute incubation at room temperature on a roller. The buffer was subsequently removed by 2-minute centrifugation at 2000 × g. Gel beads were washed five times with TE buffer containing 0.1% Tween-20. Real-time quantitative PCR (qPCR) was used to assess the proportion of bacteriophage presence after lysis. Primers specific to T4, P1, and lambda phages were utilized: T4 F: AAGCGAAAGAAGTCGGTGAA, T4 R: CGCTGTCATAGCAGCTTCAG; P1 F: CTCTGCCCGTTATTTGTGGTGT, P1 R: AGTCCGCTCGCTCTGTGTAG; and lambda F: GAAGCGTTTATGCGGAAGAG, lambda R: ACCTGCTGATCTGCGACTTA (Table S1). Gel beads were dissolved with 10 mM DTT and used as the input for qPCR. Cycling conditions included an initial denaturation at 95 °C for 10 min, followed by 40 cycles of 95 °C for 5 s and 60 °C for 45 s. Phage abundance was estimated by comparing threshold cycles (Ct) values to three standard curves generated using the known bacteriophage suspensions.

For bacterial lysis, to account for cell wall complexity, enzyme cocktails and chaotropic agents were applied. Hydrogel bead suspensions were treated with 20 ul MetaPolyzyme Multilytic Enzyme Mix (Sigma-Aldrich, no. MAC4L-5MG), then incubated on a roller at 35 °C for 6−8 h. Post-incubation, hydrogel beads were washed twice with a pre-lysis buffer (50 mM Tris-HCl, 50 mM EDTA, 0.5% Tween-20, and 0.5% Triton X-100). Hydrogel beads were then subjected to lysis in 1.25 ml of pre-lysis buffer containing 0.2 mg/ml RNase A (Invitrogen, no. 12091-021), 1 mg/ml Proteinase K (Invitrogen, no. 100005393), and 20 mg/ml lysozyme (Thermo Scientific, no. 89833), and subsequently incubated overnight at 37 °C on a roller. Complete lysis was achieved by mixing the resulting suspension with 0.35 volume of a chaotropic buffer (3M guanidine hydrochloride and 20% Tween-20) and incubating at 50 °C for 2h. To remove lysis reagents, beads were washed sequentially: twice with water containing 2% Tween-20, twice with 100% ethanol containing 0.1% Tween-20, and five times with 1× TE containing 0.1% Tween-20. All buffers used were UV-treated for at least 30 min to minimize DNA contamination. After DNA purification, fluorescent DNA staining was performed with 1× SYBR Green, and positive rates were quantified under a fluorescent microscope.

Whole-genome amplification

DNA was denatured by adding equal volumes of Buffer D (0.5 M NaOH and 1.5 M NaCl) to gel beads and incubating at room temperature for 5 min, followed by neutralization with Buffer N (0.5 M Tris-HCl and 1.5 M NaCl). Gel beads were then passed through a 40-μm cell strainer to eliminate large clumps and resuspended in 1 × CutSmart buffer (NEB, no. B7204) supplemented with 0.5% Tween-20. The gel beads suspension and MDA pre-mix solution were injected into the re-encapsulation chip at flow rates of 1.5 μl/min (microgel suspension), 3 μl/min (MDA pre-mix; Whole Genome Amplification Kit, 4basebio, no. 380100), and 8μl/min (droplet generation oil). Droplets underwent whole-genome amplification in a heating oven at 30 °C for 12 h, followed by enzyme inactivation at 70 °C for 30 min.

Debranching of whole-genome amplification products

A reaction mixture was prepared by combining 105 μl ultrapure water, 100 μl NEB buffer 2.0, 25 μl 10 mM dNTP, 50 μl T7 Endonuclease I (NEB, no. M0302L), and 20 μl Hot Start *Taq* DNA Polymerase (NEB, no. M049). The mixture was loaded into a vertically positioned 1-ml syringe backed with 200 μl HFE-7500. WGA droplets were loaded into seperate 1-ml syringe mounted vertically on a pump. Droplet merging was performed using a saltwater electrode as previously described [6]. A 2-droplet-merger was employed to combine the WGA droplet with enzyme mixture. Flow rates were set as follows: ~2 μl/min for NEB enzyme solution, 5 μl/min for NEB droplet generation oil for enzyme solution, ~2.5 μl/min for MDA droplet, and 5 μl/min for NEB droplet generation oil as spacing oil for MDA droplet. Merging was achieved using an AC potential of 850 mV at 700 Hz. The resulting emulsion was collected in a 1.5 mL tube on ice. After droplet formation, mineral oil was added, and the tube was incubated at 37 °C for 4 h for fragmentation, followed by 72 °C for 2 h for end-repair.

Barcode generation in droplets

Barcode template oligonucleotides were diluted to 4 fM in a 400 nM primers solution to minimize DNA adhesion to tube wall. A final concentration of 0.8 fM barcode template was added to a PCR mixture containing 1× Tsp buffer, 0.2 mM dNTP, 1.8 mM MgCl_2_, 2% Tween-20, 400 nM primer mixture, and 0.25 U/μl Tsp DNA polymerase (Invitrogen, no. 11448-024). The PCR solution was denatured at 90 °C for 30 s and snap-cooled for 5 min. Droplets were generated at 9 μl/min (PCR solution) and 10 μl/min (droplet generation oil), collected into PCR tubes and overlaid with mineral oil to prevent evaporation. Thermal cycling was performed under the following conditions: 90 °C for 5 s, 60 °C for 1 min (40 cycles), followed by 72 °C for 2 min, and a final hold at 12 °C. Using a fluorescent microscope, the barcode encapsulation rate was quantified using 1× SYBR Green I staining.

DNA barcoding in droplet

The 3-droplet-merger chip was used to merge droplets containing genomic DNA, barcode DNA and ligation reagents. Freshly perpared Ultra II Ligation Master Mix (NEB, no. E7648A) and enhancer (NEB, no. E4374A) were loaded into an HFE-7500-backed 1-ml syringe. After removal of mineral oil, barcode and genomic DNA droplets were also loaded into 1-ml syringes. Droplet generation oil was used for Ligation Master Mix droplet generation, while HEF-7500 served as spacing oil for barcode and genomic DNA droplets. All components were injected into the chip at the following flow rates: ~2 μl/min for ligation mixture, ~2 μl/min for genomic DNA, ~0.5 μl/min for barcode droplets and ~5 μl/min for spacing oil. Flow rates were adjusted as needed to maintain a 1:1:1 ratio of droplet merging at the chip junction. The three droplets were merged under an electric field of ~840 mV, 64.5 kHz, square wave conditions. The emulsion was collected in 1.5 mL tubes, and mineral oil was added to the emulsion before incubation on a heat block at 16 °C overnight for ligation. Emulsions were then heated to 70 °C for 10 min to inactivate the ligase.

Library preparation

In the post-barcoding step, the emulsion of droplets was broken using PFO (Sigma-Aldrich, no. 370533-25G), and the aqueous phase was collected. DNA fragments > 1000 bp were selected using 0.6 × AMPure beads (Beckman Coulter, no. A63881). Biotinylated DNA fragments were then captured using the Dynabeads kilobaseBINDER Kit (Thermo Scientific, no. 60101). Following this, damage repair and end repair were performed on the biotinylated DNA fragments using NEBNext® FFPE DNA Repair Mix (NEB, no. M6630L) and NEBNext® Ultra™ II End Repair/dA-Tailing Module (NEB, no. E7546S), respectively, with incubation at 20 °C for 30 min and 65 °C for 30 min. Subsequently, the DNA fragments with dA-tails were ligated to the second adapter using NEBNext® Ultra™ II Ligation Module (NEB, no. E7595S) at 20 °C for 90 min. Damage-repair was conducted directly without adding the second adapter for barcoded bacterial DNA. Library PCR was carried out using Platinum SuperFi II PCR Master Mix (Invitrogen, no. 12368010) and 200 nM of library-F primer, followed by purification using 0.5× AMPure beads (Beckman, no. A63881). The library concentrations were quantified using a Quantus Fluorometer, and DNA size distributions were confirmed with agarose electrophoresis. Finally, the amplified libraries were stored at -20 °C for downstream sequencing analysis.

Single Molecules, Real-Time (SMRT) sequencing

The library performed SMRT sequencing by Berry Genomics Co., Ltd. using SMRTbell ExpressTPK 2.0 kit. Briefly, the amplified DNA libraries were purified by AMPure PB beads. Then the libraries were subjected to damage- and end-repair. PacBio loop adapter was ligated to the libraries, and Endonuclease III removed the unligated DNA and extra adaptors. After being purified by AMPure PB beads, the size of the sequencing libraries was qualified by Bioanalyzer 2100 instrument. The libraries were sequenced on the PacBio Sequel II platform (Table S17).

Barcode extraction and clustering from CCS HiFi reads

Highly accurate consensus subreads (HiFi reads) were generated using the ccs (v6.0.0) with parameters “--min-passes 3 --min-rq 0.90”. The statistics of HiFi reads were summarized by seqkit [7](v0.15.0). A custom Python script was developed to extract the barcodes from the single-amplified genome (Levenshtein distances = 1 or 2). Starcode sphere clustering algorithm (v1.4) with parameter (-s) was employed to cluster the barcodes with the parameter of Levenshtein distances = 1 or 2 [8]. This clustering step is designed to collapse barcodes with PCR errors into their putative parent sequence, thus mitigating the impact of such errors on downstream analyses.

Mock community analysis

The alignment of reads from each barcode group was conducted using Minimap2 [9] (v2.22) with default parameters against the reference genomes provided by DSMZ. Barcode group purity (the fraction of reads mapping to the most mapped reference [10]) and genome coverage were calculated from reference alignment data.

Different assembly methods comparison

The reads obtained after barcode extraction were assembled using three different strategies. For direct assembly (DA), all reads were combined and assembled using SPAdes [11] (v3.13.0) with the “--sc --careful” options. For barcode-clustered assembly (BCA), the reads were first separated by their barcodes, and each barcode cluster was assembled individually with the same SPAdes settings. Finally, for Single Amplified Genome binning (SAGb), the contigs from the same barcode (presumed to originate from the same single amplified genome) were binned together. This yields one assembly per barcode cluster.

Single amplified genomes (SAGs) analysis

For single-virus analysis, contigs < 2000 bp were removed. CheckV [12] (v0.8.1) was used to assess contig completeness and contamination using default parameters. A combination of whole-genome Average Nucleotide Identity (ANI) and the alignment fraction (AF) [13] was utilized to reflect relatedness between vSAGs in this study and viral genomes from several databases, including NCBI RefSeq (updated May 2022), Metagenomic Gut Virus (MGV) [14], Gut Virome Database (GVD) [15] and Global Ocean Viromes 2.0 Dataset [16] (v2.0). FastANI [17] (v1.33) was used to calculate the ANI between all the vSAGs and the viral references. When AF > 0.6 and ANI > 95% [13], the genome was defined as a “known” species. The taxonomic assignments of SVGs were determined with Contig Annotation Tool [18] (v4.6) based on NCBI non-redundant (nr) database. The circular DNA was predicted by VIBRANT [19] (v1.2.0).

RBS classifier [20] and ViralRecall [21] (v2.0) were employed to predict potential the nucleocytoplasmic large DNA viruses (NCLDV) among the SVGs. SVGs meeting thresholds were selected as candidate NCLDVs (RBS classifier score > 1, viral recall score > 0, or the genome length > 200 kb). Subsequently, a marker gene search specific to giant viruses was conducted, with SVGs possessing two or more identified marker genes considered putative NCLDV candidates. To build a phylogenetic tree of the extended NCLDV, viral genomes with at least two out of seven marker genes were selected: D5-like helicase-primase, DNA polymerase elongation subunit family B, DNA or RNA helicases of superfamily II, packaging ATPase, RNA polymerase α subunit, RNA polymerase β subunit and YqaJ viral recombinase. The marker genes were identified against the Nucleo-Cytoplasmic Virus Orthologous Group (NCVOG) database and Giant Virus Orthologous Genes (GVOG) database with hmmsearch (v3.3.2; http://hmmer.org/) using an E-value cut-off 0.01, extracted and aligned using mafft [22] (v7.310). The phylogenetic tree was then calculated based on the concatenated alignment of all seven proteins using IQ-tree 2 [23] (v2.0.3) with ultrafast bootstrap 1000 replicates and VT + R7 model. Phylogenetic trees were visualized with iTol [24] (v5). Functional annotations on the ORFs in the viral contigs were conducted based on comparisons with the eggNOG (v5.0) database [25] using eggNOG-mapper [26] (v2).

To find crAss-like viruses in our data, Prodigal [27] (v2.6.3) was used to predict open reading frames (ORFs) with the -a and -p meta parameters. Then, using hmmscan (v3.3.0; http://hmmer.org/) and the HMM model of three marker proteins (major capsid protein, portal protein, terminase large subunit) [28] modified scripts, we searched the profiles against all protein sequences predicted in our contigs with the -T 50 parameter. SAGs containing ≥ 2 of these hallmark genes were defined as positive crAss-like viruses.

The quality of Bacterial Single Amplified Genomes (bSAGs) was assessed by CheckM [29] (v1.2.0) for completeness and contamination. A quality score was calculated as: Quality score = Completeness – 5 × Contamination [30]. The bSAGs with a quality score < 0 were removed. For medium-quality (50% ≤ completeness < 90% ) or high-quality (completeness ≥ 90%) bSAGs, FastANI [17] (v1.33) was used to calculate the average nucleotide identity (ANI) against the NCBI Bacteria Refseq database (April 2022 release). Genomes with ANI values ≥ 95% were defined as known bacteria. A phylogenetic tree was constructed based on 16S rDNA sequences. The sequences were extracted from bacterial genomes using METAXA2 [31](v2.2) and barrnap (v0.9; https://github.com/tseemann/barrnap). The 16S sequences were filtered using the following criteria: (1) length between 1000 and 1500 bp; (2) bacterial taxonomy annotation similarity > 95% at the phylum level; and (3) quality score ≥ 10. Cultural status was determined using the UHGG v2.0 database in MGnify [32], which contains 4744 representative species. For the low-completeness, ( < 50% completeness) SAGs, 16S rRNA sequences showing less than 97% similarity to the top hit in the database were considered as lack an obvious reference genome. ClustalW [33] (v2.1) was used to align the identified bacteria 16S rDNA sequences under default parameters, and alignment were manually inspected. MEGA X [34] was used for phylogenetic analyses with the Maximum Likelihood (ML) method. Annotation information was visualized by iTOL [24] (v5).

The prophage was predicted by CheckV [12] (v0.8.1) and further analyzed by VirSorter2 [35] (v2.2.3). The prophage was annotated by Contig Annotation Tool [18] using default settings and compared against the NCBI viral RefSeq protein database (v215). The bacterial taxonomy of SAGs was annotated using GTDB-TK[36] (v2.1.1). The schematic was created in part using BioRender.com.

**REFERENCES**

1. Qin, Dong, Younan Xia, George M. Whitesides. 2010. “Soft lithography for micro- and nanoscale patterning.” *Nature Protocols* 5: 491-502. <https://doi.org/10.1038/nprot.2009.234>

2. Qu, Fuyang, Shirui Zhao, Guangyao Cheng, Habibur Rahman, Qinru Xiao, Renee Wan Yi Chan, Yi-Ping Ho. 2021. “Double emulsion-pretreated microwell culture for the in vitro production of multicellular spheroids and their in situ analysis.” *Microsystems & Nanoengineering* 7: 38. <https://doi.org/10.1038/s41378-021-00267-w>

3. Zhao, Liuyang, Yu Shi, Harry Cheuk-Hay Lau, Weixin Liu, Guangwen Luo, Guoping Wang, Changan Liu, et al. 2022. “Uncovering 1058 novel human enteric DNA viruses through deep long-read third-generation sequencing and their clinical impact.” *Gastroenterology* 163: 699-711. <https://doi.org/10.1053/j.gastro.2022.05.048>

4. Patel, Anand, Rachel T. Noble, Joshua A. Steele, Michael S. Schwalbach, Ian Hewson, Jed A. Fuhrman. 2007. “Virus and prokaryote enumeration from planktonic aquatic environments by epifluorescence microscopy with SYBR Green I.” *Nature Protocols* 2: 269-276. <https://doi.org/10.1038/nprot.2007.6>

5. Mazutis, Linas, John Gilbert, W. Lloyd Ung, David A. Weitz, Andrew D. Griffiths, John A. Heyman. 2013. “Single-cell analysis and sorting using droplet-based microfluidics.” *Nature Protocols* 8: 870-891. <https://doi.org/10.1038/nprot.2013.046>

6. Sciambi, Adam, Adam R. Abate. 2014. “Generating electric fields in PDMS microfluidic devices with salt water electrodes.” *Lab on a Chip* 14: 2605-2609. <https://doi.org/10.1039/C4LC00078A>

7. Shen, Wei, Shuai Le, Yan Li, Fuquan Hu. 2016. “SeqKit: a cross-platform and ultrafast toolkit for FASTA/Q file manipulation.” *PLoS One* 11: e0163962. <https://doi.org/10.1371/journal.pone.0163962>

8. Eduard Zorita, Pol Cuscó, Guillaume J. Filion. 2015. “Starcode: sequence clustering based on all-pairs search.” *Bioinformatics,* 31:1913-1919. https://doi.org/10.1093/bioinformatics/btv053

9. Li, Heng. 2018. “Minimap2: pairwise alignment for nucleotide sequences.” *Bioinformatics* 34: 3094-3100. <https://doi.org/10.1093/bioinformatics/bty191>

10. Lan, Freeman, Benjamin Demaree, Noorsher Ahmed, Adam R. Abate. 2017. “Single-cell genome sequencing at ultra-high-throughput with microfluidic droplet barcoding.” *Nature Biotechnology* 35: 640-646. <https://doi.org/10.1038/nbt.3880>

11. Bankevich, Anton, Sergey Nurk, Dmitry Antipov, Alexey A. Gurevich, Mikhail Dvorkin, Alexander S. Kulikov, Valery M. Lesin, et al. 2012. “SPAdes: a new genome assembly algorithm and its applications to single-cell sequencing.” *Journal of Computational Biology* 19: 455-477. <https://doi.org/10.1089/cmb.2012.0021>

12. Nayfach, Stephen, Antonio Pedro Camargo, Frederik Schulz, Emiley Eloe-Fadrosh, Simon Roux, Nikos C. Kyrpides. 2021. “CheckV assesses the quality and completeness of metagenome-assembled viral genomes.” *Nature Biotechnology* 39: 578-585. <https://doi.org/10.1038/s41587-020-00774-7>

13. Varghese, Neha J., Supratim Mukherjee, Natalia Ivanova, Konstantinos T. Konstantinidis, Kostas Mavrommatis, Nikos C. Kyrpides, Amrita Pati. 2015. “Microbial species delineation using whole genome sequences.” *Nucleic Acids Research* 43: 6761-6771. <https://doi.org/10.1093/nar/gkv657>

14. Nayfach, Stephen, David Páez-Espino, Lee Call, Soo Jen Low, Hila Sberro, Natalia N. Ivanova, Amy D. Proal, et al. 2021. “Metagenomic compendium of 189,680 DNA viruses from the human gut microbiome.” *Nature microbiology* 6: 960-970. <https://doi.org/10.1038/s41564-021-00928-6>

15. Gregory, Ann C., Olivier Zablocki, Ahmed A. Zayed, Allison Howell, Benjamin Bolduc, Matthew B. Sullivan. 2020. “The gut virome database reveals age-dependent patterns of virome diversity in the human gut.” *Cell Host & Microbe* 28: 724-740.e8. <https://doi.org/10.1016/j.chom.2020.08.003>

16. Gregory, Ann C., Ahmed A. Zayed, Nádia Conceição-Neto, Ben Temperton, Ben Bolduc, Adriana Alberti, Mathieu Ardyna, et al. 2019. “Marine DNA viral macro- and microdiversity from pole to pole.” *Cell* 177: 1109-1123.e1114. <https://doi.org/10.1016/j.cell.2019.03.040>

17. Jain, Chirag, Luis M. Rodriguez-R, Adam M. Phillippy, Konstantinos T. Konstantinidis, Srinivas Aluru. 2018. “High throughput ANI analysis of 90K prokaryotic genomes reveals clear species boundaries.” *Nature Communications* 9: 5114. <https://doi.org/10.1038/s41467-018-07641-9>

18. von Meijenfeldt, F. A. Bastiaan, Ksenia Arkhipova, Diego D. Cambuy, Felipe H. Coutinho, Bas E. Dutilh. 2019. “Robust taxonomic classification of uncharted microbial sequences and bins with CAT and BAT.” *Genome Biology* 20: 217. <https://doi.org/10.1186/s13059-019-1817-x>

19. Kieft, Kristopher, Zhichao Zhou, Karthik Anantharaman. 2020. “VIBRANT: automated recovery, annotation and curation of microbial viruses, and evaluation of viral community function from genomic sequences.” *Microbiome* 8: 90. <https://doi.org/10.1186/s40168-020-00867-0>

20. Schulz, Frederik, Simon Roux, David Paez-Espino, Sean Jungbluth, David A. Walsh, Vincent J. Denef, Katherine D. McMahon, et al. 2020. “Giant virus diversity and host interactions through global metagenomics.” *Nature* 578: 432-436. <https://doi.org/10.1038/s41586-020-1957-x>

21. Aylward, Frank O., Mohammad Moniruzzaman. 2021. “ViralRecall—a flexible command-line tool for the detection of giant virus signatures in ‘omic data.” *Viruses* 13: 150. <https://www.mdpi.com/1999-4915/13/2/150>

22. Katoh, Kazutaka, Daron M. Standley. 2016. “A simple method to control over-alignment in the MAFFT multiple sequence alignment program.” *Bioinformatics* 32: 1933-1942. <https://doi.org/10.1093/bioinformatics/btw108>

23. Bui Quang Minh, Heiko A Schmidt, Olga Chernomor, Dominik Schrempf, Michael D Woodhams, Arndt von Haeseler, Robert Lanfear. 2020. “IQ-TREE 2: New Models and Efficient Methods for Phylogenetic Inference in the Genomic Era.” *Molecular biology and evolution*. 37(5): 1530-1534. https://doi.org/10.1093/molbev/msaa015

24. Letunic Ivica, and Peer Bork. 2021. “Interactive Tree Of Life (iTOL) v5: an online tool for phylogenetic tree display and annotation.” *Nucleic acids research*. 49(W1): W293-W296. https://doi.org/10.1093/nar/gkab301

25. Huerta-Cepas, Jaime, Damian Szklarczyk, Davide Heller, Ana Hernández-Plaza, Sofia K Forslund, Helen Cook, Daniel R Mende, et al. 2018. “eggNOG 5.0: a hierarchical, functionally and phylogenetically annotated orthology resource based on 5090 organisms and 2502 viruses.” *Nucleic Acids Research* 47: D309-D314. <https://doi.org/10.1093/nar/gky1085>

26. Cantalapiedra, Carlos P, Ana Hernández-Plaza, Ivica Letunic, Peer Bork, Jaime Huerta-Cepas. 2021. “eggNOG-mapper v2: functional annotation, orthology assignments, and domain prediction at the metagenomic scale.” *Molecular Biology and Evolution* 38: 5825-5829. <https://doi.org/10.1093/molbev/msab293>

27. Hyatt, Doug, Gwo-Liang Chen, Philip F. LoCascio, Miriam L. Land, Frank W. Larimer, Loren J. Hauser. 2010. “Prodigal: prokaryotic gene recognition and translation initiation site identification.” *BMC Bioinformatics* 11: 119. <https://doi.org/10.1186/1471-2105-11-119>

28. Yutin, Natalya, Sean Benler, Sergei A. Shmakov, Yuri I. Wolf, Igor Tolstoy, Mike Rayko, Dmitry Antipov, Pavel A. Pevzner, Eugene V. Koonin. 2021. “Analysis of metagenome-assembled viral genomes from the human gut reveals diverse putative CrAss-like phages with unique genomic features.” *Nature Communications* 12: 1044. <https://doi.org/10.1038/s41467-021-21350-w>

29. Parks, Donovan H, Michael Imelfort, Connor T Skennerton, Philip Hugenholtz, Gene W Tyson. 2015. “CheckM: assessing the quality of microbial genomes recovered from isolates, single cells, and metagenomes.” *Genome Research* 25: 1043-1055. <https://doi.org/10.1101/gr.186072.114>

30. Parks, Donovan H., Christian Rinke, Maria Chuvochina, Pierre-Alain Chaumeil, Ben J. Woodcroft, Paul N. Evans, Philip Hugenholtz, Gene W. Tyson. 2017. “Recovery of nearly 8,000 metagenome-assembled genomes substantially expands the tree of life.” *Nature Microbiology* 2: 1533-1542. <https://doi.org/10.1038/s41564-017-0012-7>

31. Bengtsson-Palme, Johan, Martin Hartmann, Karl Martin Eriksson, Chandan Pal, Kaisa Thorell, Dan Göran Joakim Larsson, Rolf Henrik Nilsson. 2015. “metaxa2: improved identification and taxonomic classification of small and large subunit rRNA in metagenomic data.” *Molecular Ecology Resources* 15: 1403-1414. [https://doi.org/10.1111/1755-0998.12399](https://doi.org/https://doi.org/10.1111/1755-0998.12399)

32. Almeida, Alexandre, Stephen Nayfach, Miguel Boland, Francesco Strozzi, Martin Beracochea, Zhou Jason Shi, Katherine S. Pollard, et al. 2021. “A unified catalog of 204,938 reference genomes from the human gut microbiome.” *Nature Biotechnology* 39: 105-114. <https://doi.org/10.1038/s41587-020-0603-3>

33. Larkin, M.A., G. Blackshields, N.P. Brown, R. Chenna, P.A. McGettigan, H. McWilliam, F. Valentin, et al. 2007. “Clustal W and Clustal X version 2.0.” *Bioinformatics* 23: 2947-2948. <https://doi.org/10.1093/bioinformatics/btm404>

34. Kumar, Sudhir, Glen Stecher, Michael Li, Christina Knyaz, Koichiro Tamura. 2018. “MEGA X: Molecular evolutionary genetics analysis across computing platforms.” *Molecular Biology and Evolution* 35: 1547-1549. <https://doi.org/10.1093/molbev/msy096>

35. Guo, Jiarong, Ben Bolduc, Ahmed A. Zayed, Arvind Varsani, Guillermo Dominguez-Huerta, Tom O. Delmont, Akbar Adjie Pratama, et al. 2021. “VirSorter2: a multi-classifier, expert-guided approach to detect diverse DNA and RNA viruses.” *Microbiome* 9: 37. <https://doi.org/10.1186/s40168-020-00990-y>

36. Chaumeil, Pierre-Alain, Aaron J Mussig, Philip Hugenholtz, Donovan H Parks. 2022. “GTDB-Tk v2: memory friendly classification with the genome taxonomy database.” *Bioinformatics* 38: 5315-5316. <https://doi.org/10.1093/bioinformatics/btac672>

**Figure S1 Detailed design for GSE-Seq.** (A) Schematic of the one-step droplet PCR method for barcode generation. A primer mix containing a 5'-biotinylated forward primer (Primer A), a 5'-phosphorylated reverse primer (Primer B), and a reverse primer with an additional 5' deoxyadenosine (Primer C) is used. The amplification process, involving six possible strand pairing scenarios (arrows), selectively yields a double-stranded, biotinylated barcode with a 3' thymine (T) overhang suitable for subsequent T-A ligation (highlighted by red dashed lines). *(Bottom right)* A representative fluorescence microscopy image of barcode-containing droplets stained with SYBR Green I. The observed positive droplet fraction is approximately 10%. Scale bar: 500 μm. (B) Microfluidic chip design for the initial encapsulation of single entities within a hydrogel precursor solution. (C) Microfluidic chip design for the re-encapsulation of solidified hydrogel beads, containing purified single genomes, into new droplets for whole-genome amplification (WGA). (D) Representative fluorescence microscopy images showing SYBR Green I staining of encapsulated viral (left) and bacterial (right) mock communities within hydrogel beads. The viral mock community consists of three *Escherichia coli* bacteriophages (T4, P1, and Lambda). The bacterial mock community includes *Escherichia coli*, *Staphylococcus aureus*, and *Bacillus subtilis*. Scale bar: 25 μm.

**Figure S2 Characterization of hydrogel degradation and in-droplet enzymatic reactions.** (A) Time-lapse microscopy images showing the dissolution of a single hydrogel bead over 10 minutes following the addition of 10 mM DTT. Scale bar: 50 μm. (B) Quantification of hydrogel degradation time at varying DTT concentrations. Data are presented as mean ± SEM (n=3 independent experiments). (C) Representative fluorescence microscopy images comparing the efficiency of in-droplet whole-genome amplification (WGA). The fluorescence intensity of SYBR Green I, indicating the amount of amplified DNA, is significantly higher in droplets with fully dissolved hydrogel beads (right) compared to those with undissolved beads (left). Scale bar: 100 μm. (D) Schematic of the in-droplet T-A ligation strategy. A fragmented DNA molecule with a 3' adenine (A) overhang is ligated to a double-stranded barcode with a corresponding 3' thymine (T) overhang, creating a barcoded fragment ready for the sequencing library.

**Figure S3 Quality assessment and sequencing metrics of the viral mock community.** (A) Quality and length distribution of the generated HiFi reads. (B) Distribution of the number of HiFi reads per barcode cluster, with each cluster representing a single amplified viral genome (vSAG). (C) Comparison of the relative abundance of the three phages in the mock community, as quantified by qPCR (mean ± SEM) versus the proportion of vSAGs assigned to each phage by GSE-Seq. (D) Genome coverage of GSE-Seq reads mapped to the reference genomes of three phages (T4, P1, and Lambda) in a mock community. The x-axis indicates the position along the reference genome (bp), and the y-axis represents the sequencing depth at that position.

**Figure S4 Comparison of genome assembly statistics across different methods.** (A) Distribution of assembly lengths across various assembly methods for each sample. The histograms show assembly length distributions for five samples: H1, H2, H3, and S1 (viral samples) and H4 (bacterial sample). Three assembly methods were compared: direct assembly (DA), barcode clustered assembly (BCA), and single amplified genome binning (SAGb). (B–D) Comparison of key assembly metrics for each method: (B) the length of the longest contig, (C) the total number of contigs >2 kb, and (D) the total number of contigs >10 kb. (E) Density plots comparing the distribution of genome completeness for the three assembly methods within each sample.

**Figure S5 Functional, phylogenetic, and host-linkage analysis of viral and bacterial SAGs.** (A) Three representative Nucleocytoplasmic large DNA viruses (NCLDV) single amplified genomes (SAGs) with over 100 known open reading frame (ORF) gene functions are shown. Only genes involved in metabolism, DNA replication, translation, and transcription are presented. J: Translation, ribosomal structure, and biogenesis; K: Transcription; L: Replication, recombination, and repair; C: Energy production and conversion; G: Carbohydrate transport and metabolism; E: Amino acid transport and metabolism; F: Nucleotide transport and metabolism; H: Coenzyme transport and metabolism. (B) Phylogenetic tree of medium- and high-quality bacterial single amplified genomes (bSAGs). The label indicates the barcode of each bSAG, with text color-coded according to its phylum. The first column displays the genus annotation of each bSAG, and the second column presents the ANI-based genome information for each bSAG (known ≥ 95%, novel < 95%). (C) Sankey diagram illustrating the identified prophage-host pairings. The left bar represents the taxonomic distribution of the predicted prophages at the class level. The right bar represents the distribution of their bacterial hosts at the species level. The connecting flows visualize the observed linkages between specific prophage classes and their hosts.

**Figure S6** **Microfluidic chip designs for the GSE-Seq workflow.** (A) Chip for the initial encapsulation of single entities in hydrogel droplets. (B) Chip for the re-encapsulation of solidified hydrogel beads into new droplets containing whole-genome amplification (WGA) reagents. (C) Chip for the generation of barcode droplets. (D) Two-droplet merger chip used for combining WGA droplets with droplets containing the enzymatic fragmentation mixture (T7 endonuclease I and *Taq* polymerase). (E) Three-droplet merger chip used for combining fragmented DNA droplets, barcode droplets, and ligation mixture for in-droplet barcoding. In the merger chips (D, E), the channel highlighted in red indicates the AC electrode, while the channel in blue indicates the ground electrode.
